# Supplementary figures and images for: Developmental differences in perceiving arousal and valence from dynamically unfolding emotional expressions
Source: PLoS One. 2025 Aug 8;20(8):e0329554. doi: 10.1371/journal.pone.0329554 (PMC12333977; doi:10.1371/journal.pone.0329554)

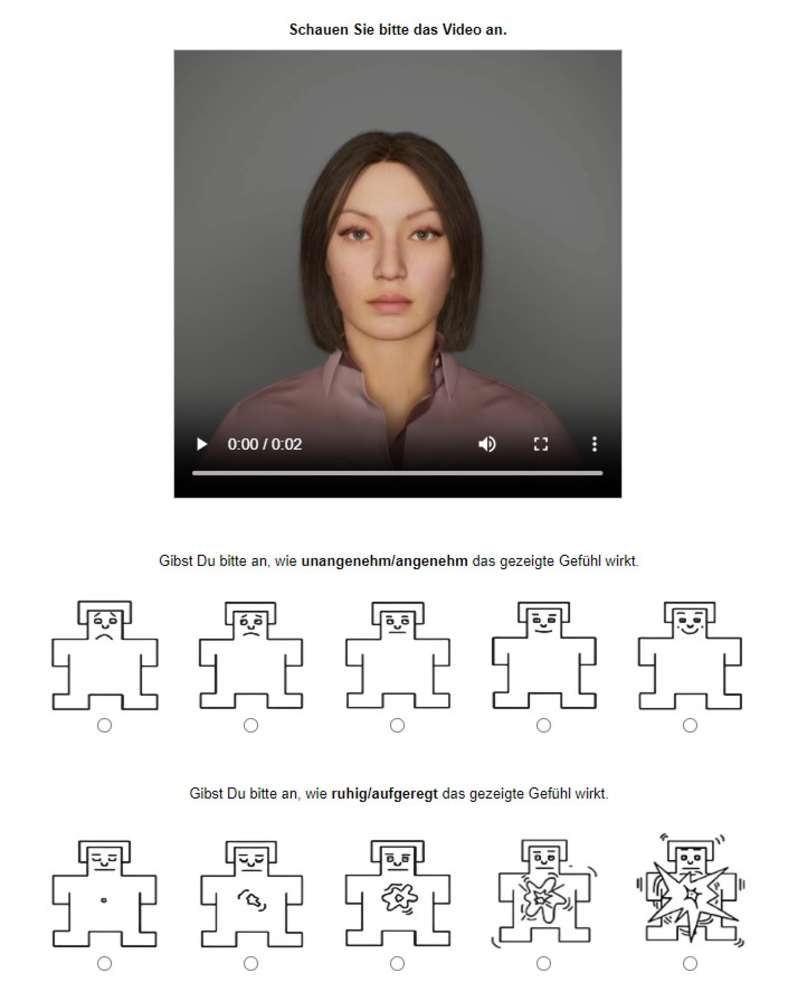

Supplement: S1_Trial example — (TIF) [file pone.0329554.s001.tif]
