# Supplementary material for: Developmental differences in perceiving arousal and valence from dynamically unfolding emotional expressions
Source: PLoS One. 2025 Aug 8;20(8):e0329554. doi: 10.1371/journal.pone.0329554 (PMC12333977; doi:10.1371/journal.pone.0329554)
Supplement: S2_Correlational heatmaps — (PDF) [file pone.0329554.s002.pdf]

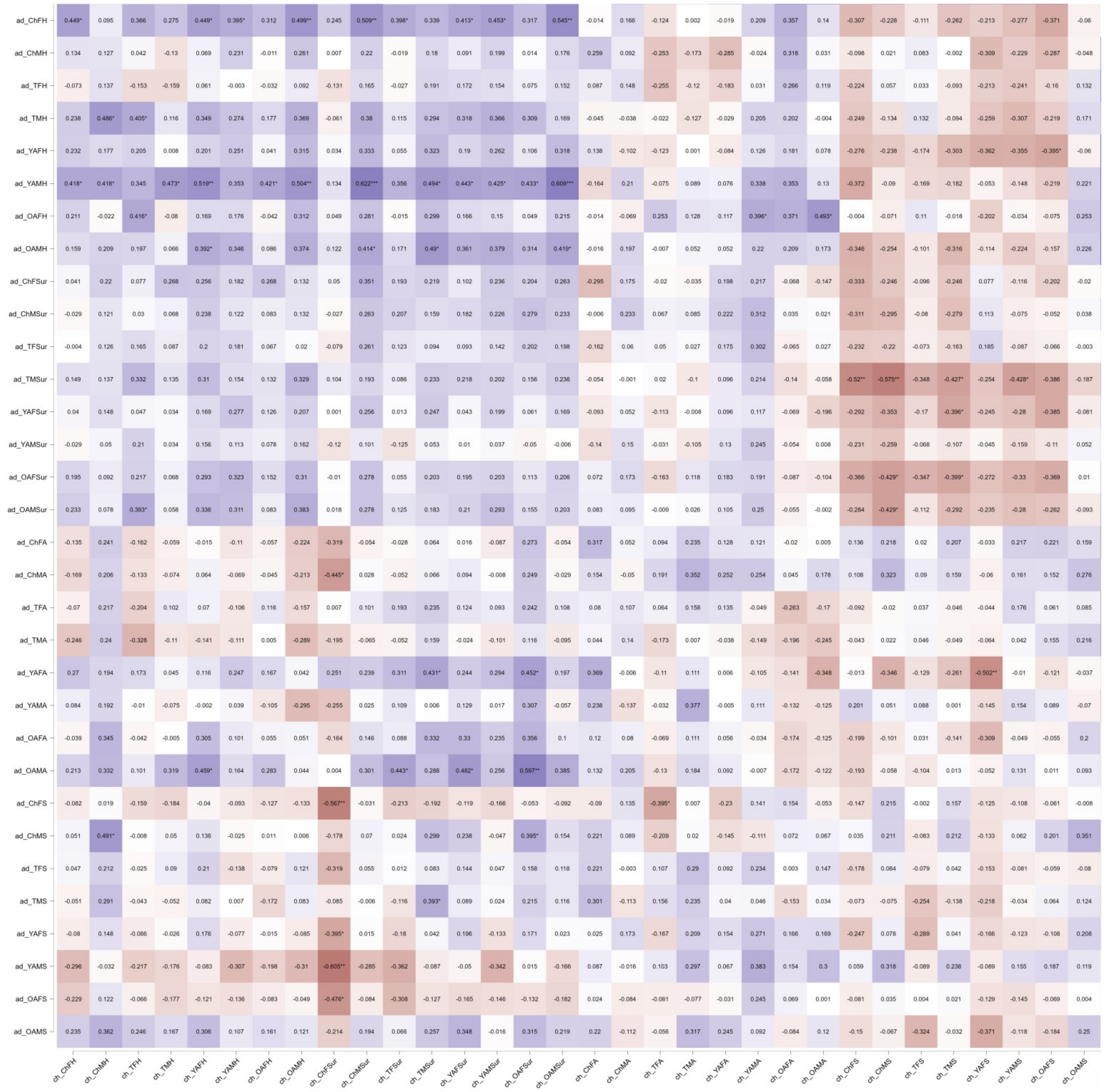

Fig 1. Correlational heatmap with the arousal responses of children and adults for the visual condition.

Legend: “ch” denotes children rating values; “ad” denotes adult rating values. Trials are abbreviated based on the age of the face (Ch = child, T = Teen, YA = Young Adult, OA = Older adults), the gender of the face (F = Female, M = Male), and the emotional expression (H = Happy, Sur = Surprised, A = Angry, S = Sad). For example, ChFH means Child Female Happy face, OAMS = Older Adult Male Sad, etc. “ch_TFA” means the response of a child participant for a Teen Female Angry face.

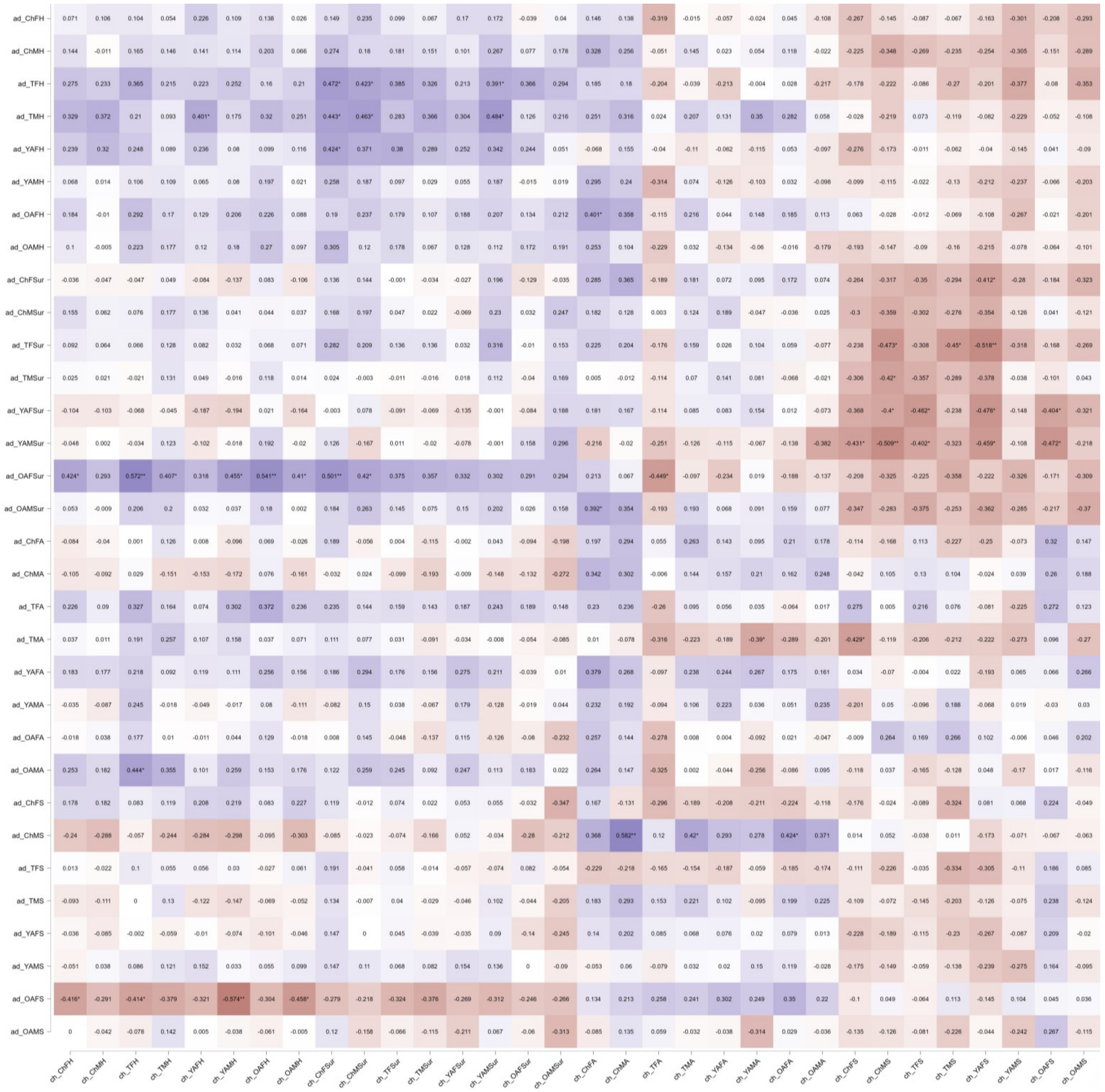

Fig 2. Correlational heatmap with the arousal responses of children and adults for the visual-verbal condition.

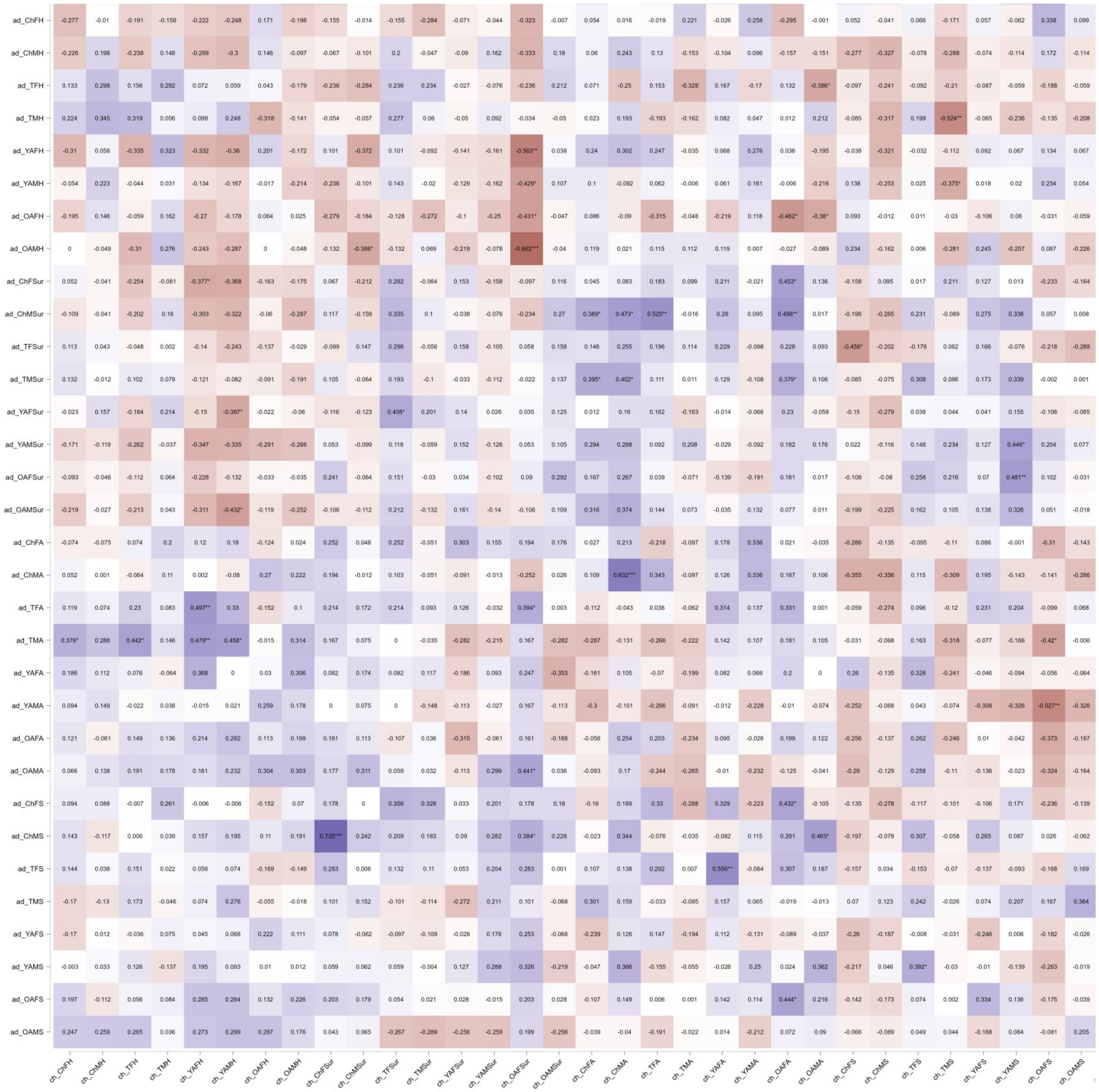

Fig 3. Correlational heatmap with the valence responses of children and adults for the visual condition.

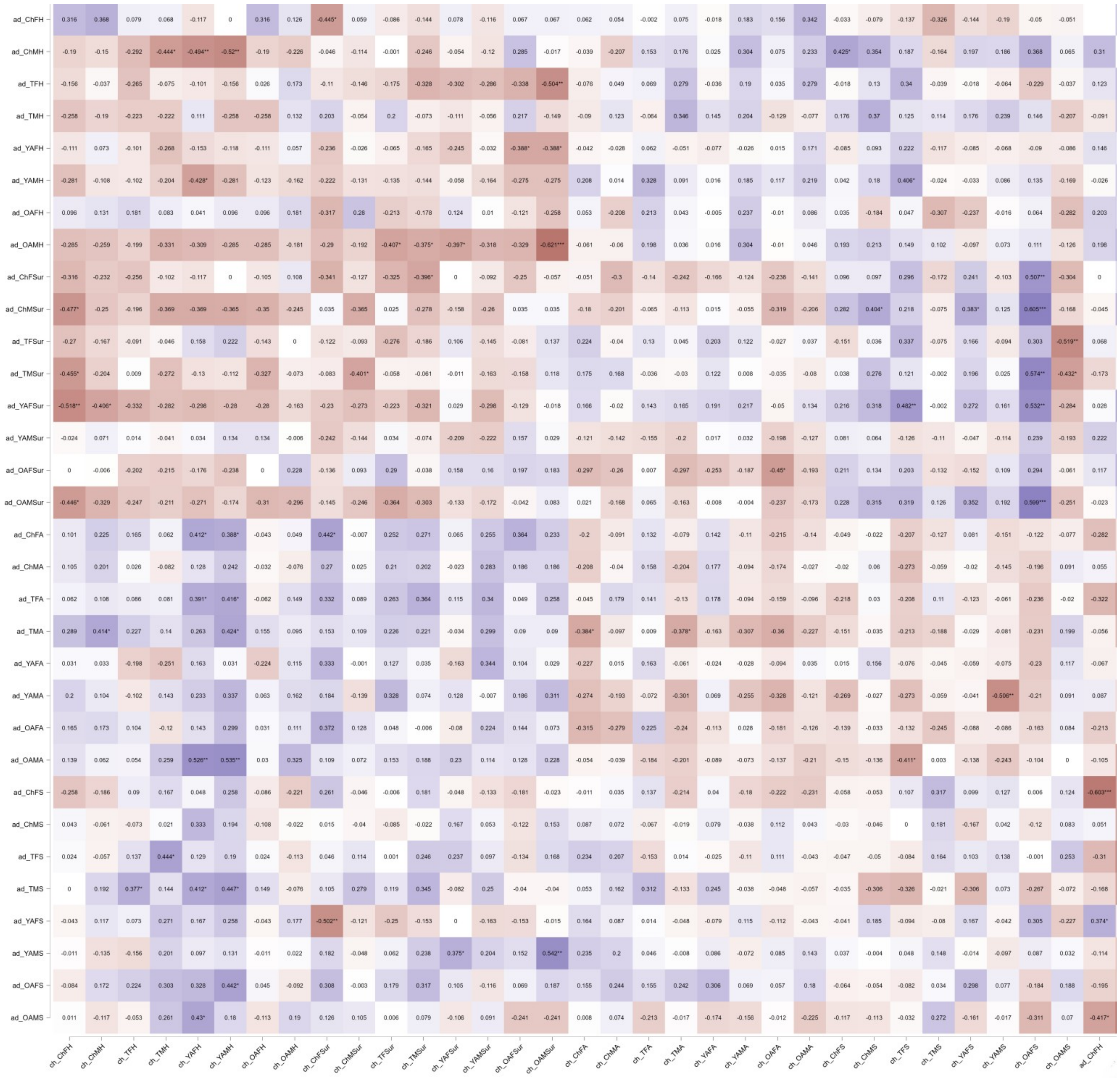

Fig 4. Correlational heatmap with the valence responses of children and adults for the visual-verbal condition.
