# Supplementary material for: Developmental differences in perceiving arousal and valence from dynamically unfolding emotional expressions
Source: PLoS One. 2025 Aug 8;20(8):e0329554. doi: 10.1371/journal.pone.0329554 (PMC12333977; doi:10.1371/journal.pone.0329554)
Supplement: Attachment — Submitted filename: Response to Reviewers.docx [file pone.0329554.s004.docx]

Dear Mr. Cruz-Albarran,

Thank you very much for evaluating our manuscript and giving us the opportunity to revise it. Attached please find the revised version of the manuscript and our detailed replies to each of the concerns raised by the reviewers.

We have carefully responded to all of the suggestions and, for example, restructured the Introduction for better flow and coherence as suggested by the reviewers, and added a Future Directions subheading in the Discussion to address the applications of our findings.

We would like to thank you and all three reviewers for the helpful comments and the overall positive feedback.

We hope that our revision satisfactorily addresses all concerns and look forward to your decision.

Sincerely,

Nikol Tsenkova

On behalf of all authors

Replies to Reviewer 1

1. An in-text citation for the ‘numerous studies’ referenced in line 55 would help direct readers to the most relevant studies and/or reviews.

Thank you for this observation. In the revised manuscript, we have added additional references to support the statement in line 55 (original), which now appear in lines 54-56.
“Consequently, numerous studies have focused on exploring the developmental trajectory of emotion perception across various age groups – mostly in children [5-6] and adults [7], but also in adolescents [8-9], and older adults [10].”

2. Across lines 132-135, the authors explain that the valence of the bias induced during emotion recognition tasks (positive vs. negative) is task dependent. Given that the previous paragraphs detail age-dependent differences in positivity/negativity biases, it would be helpful if the authors could clarify whether of not these task-specific biases show any age-dependence or otherwise have the same effects upon children as adults.

Thank you for this insightful comment. We have clarified whether these findings discussed in lines 132-135 (original) are in children, adults or both age groups (now in lines 81-85).
While some tasks are more likely to produce a positivity bias (e.g., identification tasks: children [19], adults [20]; intensity and arousal ratings: children [21]), others have shown to induce a negativity bias (e.g., visual search tasks: both children and adults [22]; recognition tasks: younger and older adults [23]; for a comprehensive review on infants and children, see [24, 25]).

3. Lines 169-170 describe how the dependent variables were split across groups for children but not adults. Including a line on the rationale for this split would aid clarity.

We fully agree with the reviewer's suggestion and have now added an additional explanation for our decision to split the children groups (lines 154-158 in revised manuscript).
“We tested two separate groups of German children, one for each of the two dependent measures (arousal and valence), and one group of adults who performed both measures simultaneously. In order to avoid fatigue and confusion regarding the difference between the scales, we separated the children sample into two groups. We did not expect such issues with the adult sample, hence there was only one adult group.”

4. Captions for figures 4 and 5 would benefit from a note to define what asterisks represent (e.g., * = p < .05).

Thank you, we have now added that.

5. Missing close bracket on reference 32, line 571.

Thank you for bringing this to our attention. It is now fixed!

6. Regarding the evolutionary explanation across lines 578-581, where it is stated that children’s negativity bias is driven by heightened threat detections due to their relative vulnerability: Whilst I find this a plausible suggestion, in the absence of specifically testing this hypothesis – or indeed referencing work to support this notion – the authors may wish to consider the addition of an alternative, perhaps non-evolutionary, explanation for this finding. In its current form, I am concerned that the explanation is vulnerable to the ‘just so’ critique of evolutionary explanations in psychology.

Thank you for highlighting this crucial point. We have addressed this issue by citing several works that support our observations and have improved the paragraph between lines 573-588.
“Additionally, we observed that anger, not sadness, elicited higher ratings in arousal, which is in line with previous research [11]. Based on this, we hypothesize one possible explanation for our findings. As discussed by Vaish [24] in their literature review, infants may attend to negative faces (such as anger or fear) more frequently than happy ones, given that these emotions signal potential danger, which is more relevant to the infant. From an evolutionarily perspective, we suspect that children, being less capable of defending themselves, could be more reactive to expressions of anger due to the perceived higher threat value. Interestingly, a similar framework was proposed by Vesker and team [14], where they found a positivity bias for arousal from static images. They suggest that children might seek positive information from their surrounding as a means of protection from threats. While their findings revealed a positivity bias, both studies align with the evolutionary mechanisms driving these biases, although the direction may vary based on the stimulus material. Considering that our stimuli, we suspect that the difference in the observed biases in our study and Vesker’s study [14] could be attributed to the nature of our stimuli – dynamically unfolding and containing verbal information that might have enhanced the perception of emotions for both age groups due to the additional cues from the voice (e.g. an angry/sad tone).”

Moreover, would the logic presented here not also dictate that angry expressions in physically imposing individuals (e.g., a male adult) should be perceived as more arousing to children than the angry expressions of less physically imposing individuals (e.g., a female child)? I note that the three-way interaction between Age of Faces, Valence Category, and Stimulus Type (described across lines 396-400) seemingly revealed no significant difference in young adults compared to other age groups for negative valence.

Regarding the comment on suspected higher arousal for angry adult faces compared to angry children faces, we decided to run an additional analysis with the few trials featuring angry faces. We performed a paired-samples *t*-test to determine whether children participants found adult angry faces as more arousing compared to children’s angry faces. However, we found no significant difference, *t*(25) = -0.62, p = .53. It is important to note that we only had four trials for each group (adult angry faces and child angry faces), which may have limited the statistical power of this analysis. With a larger number of trials, it could be possible to better explore this potential effect.

7. Further discussion of implications of this study would strengthen the manuscript. The authors do discuss the implications of their findings in relation to the validation of a new stimulus database – and indeed, this is a great strength of the study – however, currently lacking is further discussion of the findings’ real-world implications. For example, through lines 612 and 613, the authors report that ‘The results from both age groups suggest inherent differences in the processing of specific emotions in terms of valence.’ What are the broader implications of this knowledge on the ontogeny of emotion processing? The authors may wish to ground their findings in potential real-world applications (be that in educational settings, creating media, or parenting etc.)

We are extremely grateful for this suggestion and have made the necessary changes by introducing a new subheading within Discussion, entitled “Future Directions” (lines 651-690). We first discuss the importance of employing ecologically valid stimuli for exploring developmental differences in emotion perception (lines 651-657). Then we address the effectiveness of using digital avatars for such endeavors (lines 658-665). Furthermore, we highlight several studies that have successfully implemented digital avatars in areas like teaching people with autism social skills, aiding people with Attention Deficit Hyperactivity Disorder with their overall performance, as well as serving well in educational settings (lines 666-672). Lastly, and most importantly, we underscore the application of our findings in terms of teaching and parenting styles implementing positive reinforcement, as well as the importance of reframing negative experiences to crucial for developing important life skills in youth programs and in early childhood education and care practices (lines 673-690).

Replies to Reviewer 2

Abstract
Line 40-41, the authors said “our findings partially align with previous research…”, the statement “previous research” is not entirely appropriate in Abstract because it is too broad for readers. Specifically, it is difficult for readers to have a clear understanding of the term “previous research” when it first appeared in Abstract section.

Thank you for your suggestion, we have now removed that!

Keywords
The word “development” is not suitable as a keyword, and it is recommended to select a suitable one again.

We acknowledge that the keyword “development” is too general and thus have changed it to “emotional development” to be more specific and in line with the manuscript’s topic.

Introduction
The structure and presentation of this section are not clear. It is recommended to reorganize the new structure based on the research topic and content. Overall, the introduction section needs a clear main line to gradually induce the purpose, significance, and innovation of the current research.

Specifically, Line 51-61, this section is too short and there is too little preparation in this section. It is recommended to provide necessary research background and add necessary connecting paragraphs to continue the context.

It is necessary to elaborate on the research progress related to emotion perception in detail, in order to reasonably introduce the innovation of current study.

It is necessary to clearly indicate the new contributions and values of current research in this field or related research areas.

Line 63 Naturalness of emotional stimuli in prior studies
Line 107 Development of emotion perception
Line 137 Usage of Metahumans

Do the above three parts belong to the literature review? If so, please provide a clear structural explanation, for example adding a new section literature review. If not, the necessary explanations should also be provided to facilitate readers' better understanding of the authors' work. In addition, for each individual part, the authors should provide necessary summaries rather than just comments.

We completely agree with the reviewer’s observation and are grateful for the suggestions. To address this, we have restructured the introduction in several ways.

For clarity, we have removed the subheadings and instead have focused on bridging the separate parts of the introduction with summaries and connecting sentences. Additionally, we have rearranged certain parts. We first start with an overview of developmental changes in emotional development (line 51 to 70), report observed biases in the literature on developmental emotion perception (line 70 to 85), underscore reasons for the issue of inconsistent bias findings and report studies that have addressed these issues (line 86 to 122), and finally, propose a new solution for exploring age-related differences in emotion perception, i.e. by employing digital avatars (line 123 to 141). We conclude with the aims of this study (line 142 to 148).

Methods
Line 168 Participants, in this study, the number of participants in each group seems to be very small. Can this small sample size meet the requirements of data statistics? How to ensure the reliability and applicability of data results?

Thank you for suggesting this clarification. We have mentioned our a priori power analysis in lines 175 and 180, where we calculated that a total of 36 participants would be enough to test our hypothesis. We have collected data from a total of 82 participants (28 adults for both measures, 26 children for arousal, and 28 children for valence). Additionally, we have done post hoc power analyses found in lines 418-420 (for arousal):
“We performed a post-hoc G-Power analysis for arousal (8 measurements, 54 participants) revealed observed power of 71.9%, slightly below the conventional threshold of .80.”

And in lines 507-509 (for valence):
“We performed a post-hoc G-Power analysis (4 measurements, 56 participants) which revealed observed power of 93.1%, indicating ample power for this measure.”

Line 266-267, the authors said “three additional scales”, Have the authors conducted a reliability and validity test of these scales?

Thank you for bringing this to our attention. We have now included a thorough explanation of the scales and several citations showing the validity and reliability of the mentioned scales (line 264-271):
“The Godspeed Questionnaire (GQ) is the most frequently used tool in the field of Human-Robot Interaction [65], with over 3,000 citations as of April 2025, and has been translated into 19 languages. It consists of five scales, which can be used independently: Anthropomorphism (α = 0.87), Animacy (α = 0.92), Likeability (α = 0.70), Perceived Intelligence (α = 0.75), and Perceived Safety (α = 0.91) [66]. The German version of the GQ [67] has been reported to have good internal reliability (α = 0.70). In terms of validity, only the Polish version [68] has undergone factor analysis, which yielded a total variance of 74.24% for the four-factor solution.”

Results
Line 306-307, although the authors stated that gender differences are not significant, it is best to present data results to support it. In fact, gender is a good factor worth exploring the difference between them.

Thank you for this comment. We have now added this information in lines 311-314.
“Furthermore, we found no significant gender effects for either of the two measures – for arousal, F(1, 50) = .11, p = .73, η2p = .002, for valence, F(1, 52) = .23, p = .63, η2p = .004. Thus, we did not include gender in all further analyses.”

Line 314, The writing of “t” in the expression of “t-tests” should be italicized, namely, “t-tests”

Thank you, we have now changed this.

Line 357, the authors were advised to try changing the presentation format of Figure 2, 3. 5, and 6, such as replacing the current wireframe with a correlation heatmap (including correlation coefficients).

Thank you for your insightful suggestion! We have added four correlational heatmaps as Supplementary Material, showing the specific correlational coefficient of the responses between children and adults for each stimulus. We could not include them in the manuscript due to the large size of the correlational heatmap and considered it would be best to have this information in Supplementary Material instead.

Discussion
Line 572-576, the authors' discussion are suggested to cite necessary references to support the analysis, thereby enhancing the reliability of the discussion.

Thank you for your suggestion; we have now added a reference to support our observations (lines 569-572).

Line 601, “aligns with previous findings [32]”, the cited reference is the same as those in line 571. It is recommended that the authors search for new relevant literature to support discussion and avoid repeatedly citing the same article.

Thank you for this suggestion. We acknowledge the repeated citation and have now included an additional study by the same author, which - although based on a different task - shares important similarities with the design and goals of our own experiment. It was challenging to find further relevant literature, considering the limited number of studies that specifically examine arousal and valence ratings across both children and adults, particularly in a non-correlational context. Considering our study’s design and research questions which are closely aligned with the original paper cited, and given that it is, to our knowledge, the only one addressing these specific questions, we focused our comparison primarily on that work.

Line 602-604, the authors' discussion are suggested to cite necessary references to support the analysis, thereby enhancing the reliability of the discussion.

Thank you for this suggestion. Currently, we are not aware of studies in which children have rated the valence of dynamically presented happy and happy-surprised expressions. As such, we can only offer a tentative interpretation of our findings. Since both emotions were constructed to include overlapping facial features—particularly the presence of a smile—it may have contributed to the similar valence ratings observed, making differences between the two expressions less likely.

Line 634-637, the authors' discussion are suggested to cite necessary references to support the analysis, thereby enhancing the reliability of the discussion.

We really appreciate you for bringing our attention to this detail. To improve the lack of support for our conclusions, we have added several new citations supporting our findings (line 638-648):
“The only notable difference observed was in perceived realism, with adults rating the avatars as more realistic than children did. This discrepancy may be related to differences in media exposure and realism familiarity. Adults are generally more accustomed to lifelike digital representations through experiences with video games, films, and other forms of digital media. In contrast, children are typically exposed to more stylized, caricatured animations, which could influence their standard of realism. Supporting this idea, gaming statistics from 2018 [72] indicated that the average age of gamers was 34, with only 17% under the age of 18—suggesting that adults are more frequently engaged with realistic digital environments. Additionally, research has shown that adults tend to prefer realistic over stylized characters [73], whereas children often favor cartoon-like presenters in educational content [74].”

Conclusions
Line 660, “likely due to”, it is not appropriate to use the term 'due to' in the conclusion section, because it is widely used in discussion section. The conclusion section should be clear research findings obtained, rather than uncertain or ambiguous statements.

We completely agree with this suggestion and have removed it from our Conclusion section.

References
Some of the cited references are too outdated. It is recommended to update or supplement with new references.

Thank you for this recommendation. We have now added several more references from recent years, more notably two meta-analyses on the topic of emotion perception development in adults (Hayes, 2020) and in children (Riddell, 2024).

Figures
The presentation of graphics should be improved to enhance their aesthetic appeal and clarity.

Thank you for your feedback. We acknowledge the suggestion regarding the graphical presentation. Our intention was to maintain a minimalistic and clean design to emphasize clarity and ease of interpretation, in line with the visual style observed in several related studies. We believe this approach allows the main findings to stand out without introducing unnecessary visual complexity.

Replies to Reviewer 3

Comment #1: Despite the significant interest and value of the article, the introduction presents opportunities for improvement, particularly in terms of its organization. Better structuring of this section could lead to a more fluid and coherent reading experience, allowing readers to quickly grasp the research problem, study objectives, and relevance within the field. The introduction could include a clearer exposition of the research gap this study aims to fill, along with a more structured argumentation regarding its importance and innovation, always grounded in previous similar studies.

We are very grateful for the recommendations. In an attempt to improve the introduction, we have restructured it in several ways.
For clarity, we have removed the subheadings and instead have focused on bridging the separate parts of the introduction with summaries and connecting sentences. Additionally, we have rearranged certain parts. We first start with an overview of developmental changes in emotional development (line 51 to 70), report observed biases in the literature on developmental emotion perception (line 70 to 85), underscore reasons for the issue of inconsistent bias findings and report studies that have addressed these issues (line 86 to 122), and finally, propose a new solution for exploring age-related differences in emotion perception, i.e. by employing digital avatars (line 123 to 141). We conclude with the aims of this study (line 142 to 148).

Comment #2: The methodological description could be more detailed, particularly regarding the participant recruitment process and the type of sampling used. These details are crucial to ensuring research transparency, allowing other researchers to understand and potentially replicate the study. A more precise and rigorous description of the employed method would strengthen the credibility of the results and help justify the methodological choices made throughout the research.

Thank you for your insightful comment and suggestions. We have addressed these issues by adding a more detailed explanation to Methods section regarding the participants (lines 163-168):
“Children were recruited via the contact database of the Department of Developmental Psychology at the Justus Liebig University Giessen. This database consists of children of all ages (from infancy to adolescence), whose parents have given their consent to be contacted for experiments done in the Department of Developmental Psychology. The specific lists from which participants were contacted were children born in the months between May 2017 and February 2018, aged 6 and 7 years.”

Comment #3: Regarding data presentation, the graphs used are well-constructed and visually clear, facilitating the understanding of the results. However, it would be beneficial if these representations were accompanied by a more in-depth analysis, highlighting their relevance within the research context and providing detailed interpretations of what the data reveal. The inclusion of comparisons with other studies or more extensive explanations could further enrich this section.

Thank you for your helpful feedback. We have now added a short explanatory sentence before each analysis and a summarizing sentence for clarity and ease of reading.

Comment #4: Another point that deserves discussion is the designation of so-called "negative emotions." While this term is widely used in the literature, it would be worth questioning its appropriateness. Is the categorization of emotions as "negative" the most suitable? Is there a solid theoretical foundation that justifies this terminology? If not, adopting an alternative nomenclature (such as "unpleasant emotions") or at least discussing the implications of using this concept in the study’s field could be more productive.

Thank you for your thoughtful comment. We acknowledge that the term "negative emotions" is widely used in the literature and has become a standard way to describe emotions that are typically associated with discomfort, distress, or unpleasant experiences. In the context of our study, we use "negative" not to carry any personal or evaluative judgment, but rather as a convenient shorthand for emotions that are generally perceived as less pleasant, much like "positive emotions" are used to refer to those that are typically pleasurable. While we agree that exploring alternative terminology, such as "unpleasant emotions," could be beneficial for clarity, we chose to use "negative" because it aligns with much of the existing literature and theoretical frameworks in this field. For instance, our rating scales are based on Russell’s Circumplex Model (1980) where the valence axis is described using the “positive-negative” terminology, while the construction of our stimuli is based on Paul Ekman’s Basic Emotion Theory and the FACS (1978) which also borrows the “positive-negative” terminology.

Comment #5: Finally, an essential aspect to address is the comparability of this study with previous research. To what extent do the innovations brought by this work allow direct comparisons with other published studies? The study could benefit from a deeper analysis of how it positions itself within the existing scientific landscape, highlighting similarities and differences concerning prior research. This type of discussion could help contextualize the findings more effectively and reinforce the originality of the research.

We completely agree with this recommendation and have made the necessary revisions by adding a separate subheading in Discussion, entitled “Future Directions” (lines 652-692). We first discuss the importance of employing ecologically valid stimuli for exploring developmental differences in emotion perception (lines 651-657). Then we address the effectiveness of using digital avatars for such endeavors (lines 658-665). Furthermore, we highlight several studies that have successfully implemented digital avatars in areas like teaching people with autism social skills, aiding people with Attention Deficit Hyperactivity Disorder with their overall performance, as well as serving well in educational settings (lines 666-672). Lastly, and most importantly, we underscore the application of our findings in terms of teaching and parenting styles implementing positive reinforcement, as well as the importance of reframing negative experiences to crucial for developing important life skills in youth programs and in early childhood education and care practices (lines 673-690).

We would like to thank the reviewers for all the valuable comments that greatly improved the manuscript.
