# Supplementary material for: Developmental differences in perceiving arousal and valence from dynamically unfolding emotional expressions
Source: PLoS One. 2025 Aug 8;20(8):e0329554. doi: 10.1371/journal.pone.0329554 (PMC12333977; doi:10.1371/journal.pone.0329554)
Supplement: Attachment — Submitted filename: Response to Reviewers #2.docx [file pone.0329554.s005.docx]

Dear Mr. Cruz-Albarran,

Thank you very much for evaluating our manuscript and giving us the opportunity to revise it. Attached please find the revised version of the manuscript and our detailed replies to the last reviewer.

We would like to thank you and all reviewers for the helpful comments and the overall positive feedback.

We hope that our revision satisfactorily addresses all concerns and look forward to your decision.

Sincerely,

Nikol Tsenkova

On behalf of all authors

Response to Reviewer #4:

1. My first comment refers to consent; you state that parents gave consent for their children, but were children asked if they wanted to take part? At that age they would have been able to understand and give assent. Similarly, were children and young people able to give consent for their voices to be used for the vocalisations, or did parents give this consent too?

Thank you for this important comment. Since the study was conducted online, parents were instructed to involve their children only if they were willing and comfortable taking part. In two cases, children declined participation, which we fully respected.

Regarding the voice recordings, parents again obtained verbal assent from their children, who were informed about the purpose and nature of the recordings. All recordings were made with the children's full knowledge and agreement.

2. My second comments is around payment, why did child participants get paid less than the adults?

Thank you for the question. The payment difference reflects the shorter duration of the task for children. They rated only half the videos using one scale (about 30 minutes total), whereas adults completed both scales across the full set of 128 videos, which took significantly longer.

3. Thirdly, I was wondering if the race of the avatars made any difference to the perceptions by participants. I know there is some evidence people are better at recognising emotions from those with a similar ethnic background, was this analysis carried out/possible?

Thank you for this insightful comment. We considered this aspect during the creation of our database, but as the current study did not aim to examine race-related effects, we chose to use only Caucasian avatars to maintain consistency and reduce variability. As such, no analysis on racial bias was possible. However, we plan to expand the database to include more diverse facial representations in future work.

4. Finally, autistic communities prefer 'autistic' rather than 'with autism', using this language would bring the terminology up to date and in line with the autistic people's preferences.

This is a very helpful comment, it’s much appreciated! We have now changed it as you have suggested.

We would like to thank the reviewer for all the valuable comments and suggestions that improved our manuscript.
